# Rheumatic manifestations of COVID-19: a systematic review and meta-analysis

**DOI:** 10.1186/s41927-020-00165-0

**Published:** 2020-10-28

**Authors:** Jacopo Ciaffi, Riccardo Meliconi, Piero Ruscitti, Onorina Berardicurti, Roberto Giacomelli, Francesco Ursini

**Affiliations:** 1 Medicine & Rheumatology Unit, IRCCS Istituto Ortopedico Rizzoli (IOR), via Pupilli 1, 40136 Bologna, Italy; 2 Department of Biomedical and Neuromotor Sciences (DIBINEM), Section of Rheumatology, University of Bologna, Bologna, Italy; 3 Rheumatology Unit, Department of Biotechnological and Applied Clinical Sciences, University of L’Aquila, L’Aquila, Italy

**Keywords:** COVID-19, Arthralgia, Myalgia, Fatigue, Rheumatology

## Abstract

**Background:**

Different proportions of musculoskeletal or autoimmune manifestations associated with COVID-19 have been reported in literature. We performed a systematic review and meta-analysis with the aim of assessing the prevalence of rheumatic manifestations in patients affected by COVID-19, as initial symptom or during disease course.

**Methods:**

A database search was run on May 18th, 2020, using two distinct strategies. We were interested in the percentage of symptoms of potential rheumatologic interest observed in large population studies of COVID-19 cases, and in identifying uncommon autoimmune disorders described in patients with COVID-19. For manifestations individually reported, a meta-analysis was performed taking into consideration the proportion of COVID-19 patients presenting the symptom.

**Results:**

Eighty eight original articles were included in the systematic review and 51 in the meta-analysis. We found pooled estimates of 19% for muscle pain and 32% for fatigue as initial symptom of COVID-19 presentation and, respectively, of 16 and 36% during the disease course. Only one article discussed arthralgia as unique symptom. Additionally, we found that vasculitis, chilblains, presence of autoantibodies commonly found in patients with rheumatic diseases, or autoimmune haematological and neurological disorders have all been reported in patients with COVID-19.

**Conclusions:**

In conclusion, our review and meta-analysis emphasises that symptoms potentially leading to rheumatologic referral are common in patients with COVID-19. Therefore, COVID-19 is a new differential diagnosis to bear in mind when evaluating patients with musculoskeletal symptoms and rheumatologists might play a crucial role in identifying COVID-19 cases in early phases of the illness.

## Background

At the end of 2019 several cases of atypical pneumonia emerged in the Chinese province of Hubei and, in January 2020, severe acute respiratory virus 2 (SARS-CoV-2) was identified as the causative agent of the novel coronavirus disease 2019 (COVID-19) [1], representing the third major coronavirus infection of the twenty-first century after severe acute respiratory syndrome (SARS) [2] and Middle East respiratory syndrome (MERS) [3]. As of August 19, 2020, 22.173.973 confirmed cases and 781.756 deaths were reported globally (https://coronavirus.jhu.edu/map.html). Spreading from country to country, COVID-19 rapidly became a key priority for the whole scientific community. Growing and compelling evidence suggests that the manifestations of COVID-19 are protean, ranging from laboratory-confirmed asymptomatic infection to critical illness with rapidly progressive respiratory distress syndrome [4]. In symptomatic cases, current literature outlines how respiratory and constitutional symptoms are frequently reported [4]. The finding of signs and symptoms of COVID-19 extending beyond the respiratory tract can be explained, at least in part, by the ubiquitous expression and tissue distribution of angiotensin-converting enzyme 2 (ACE2), the major SARS-CoV-2 entry receptor [5]. In particular, ACE-2 is found also in bowel, endothelium of small vessels, smooth muscle, skeletal muscle and even synovial tissue [6]. It is therefore not unexpected that, besides cough and dyspnoea, COVID-19 patients often experience fever, fatigue, muscle pain, or arthralgia. However, some of the symptoms caused by COVID-19 are commonly described in other diseases and are frequently reported, for instance, also in patients with rheumatic conditions. Interestingly, the association between viral infections and rheumatic diseases is already well-recognised. Viruses can be direct etiologic agents of acute and chronic arthritis [7], and of different forms of vasculitis, both in children and adults [8]. Moreover, a role in the pathogenesis of systemic sclerosis [9] and of polymyalgia rheumatica or giant-cell arteritis [10] has been proposed. Although the causal relationship between a viral trigger and rheumatic diseases is well-known to rheumatologists, when patients with musculoskeletal complaints are evaluated, the identification of an infectious aetiology can be extremely complicated as findings are often equivocal. Since symptoms of potential rheumatologic interest have been frequently reported in COVID-19 patients, the new virus outbreak represents a previously unseen differential diagnosis to be henceforth taken into consideration. However, inconsistent percentages of musculoskeletal symptoms are reported in literature. The aim of the present systematic review and meta-analysis is to provide an updated estimate of the prevalence of clinical manifestations of potential rheumatologic relevance in COVID-19, emphasising how rheumatologists might play a crucial role in identifying cases of COVID-19 presenting with extra-respiratory symptoms.

## Methods

### Search strategy and study selection

The systematic review was performed on MedLine through PubMed search. Two search strings were built. The first string was primarily aimed at identifying large cohort studies or randomised controlled trials (RCTs) reporting clinical characteristics of patients affected by COVID-19. Additionally, to ensure no relevant references describing manifestations of potential rheumatologic interest were missed, we ran a second search using specific keywords referring to rheumatic symptoms possibly related to COVID-19, as suggested by a preliminary appraisal of currently available evidence.

First search string was: (“cohort” or “observational” or “retrospective” or “prospective” or “trial” or “cross-sectional”) and (“covid*” or “sars-cov-2” or “novel coronavirus” or “2019-ncov”) and (“symptom*” or “clinical features” or “clinical characteristic*”).

Second search string was: (“vasculitis” or “ulcer*” or “raynaud*” or “arthritis” or “acrocyanosis” or “chilblains” or “kawasaki” or “autoimmun*” or “autoantibodies” or “ana” or “anti nuclear” or “antiphospholipid” or “anca” or “citrullinated” or “rheumatoid factor”) and (“covid*” or “sars-cov-2” or “novel coronavirus” or “2019-ncov”).

No date restriction was applied and two investigators (J.C. and R.M.) worked independently to screen titles and abstracts of the literature retrieved up to 18th May 2020. Full-text evaluation was then performed, along with manual search of references to identify additional relevant papers. Disagreements were resolved through discussion with a third investigator (F.U.) when consensus could not be achieved. In drafting the final manuscript, we followed the Preferred Reporting Items for Systematic Reviews and Meta-Analyses (PRISMA) guidelines [11].

### Eligibility criteria

For the first search, on the basis of preliminary scouting of literature, we hypothesized a prevalence of musculoskeletal symptoms in COVID-19 of about 20%. Accordingly, we calculated a minimum sample size of 106 patients to estimate such proportion with 5% absolute precision and 80% confidence. On this basis, we decided to include in our systematic review and meta-analysis only studies reporting cohorts of at least 100 patients. The PICO (population, intervention, comparator, outcome) framework [12] was applied to build the search question and the inclusion/exclusion criteria. Publications written in a language other than English were excluded. All studies, published as peer-reviewed final articles and meeting the following criteria, were considered eligible:

- Population: children or adult patients with diagnosis of COVID-19;
- Intervention: assessment of clinical characteristics at onset of COVID-19 or during its evolution;
- Comparator: the presence of a comparator was not considered necessary;
- Outcomes: percentage of patients presenting symptoms of potential rheumatologic interest.

### Data extraction and quality assessment

Data were extracted and summarized by the first author (J.C.) and revised by the second author (R.M.). From each selected article, the following features were reported: first author; year of publication; origin; study design; total number of patients; hospital or non-hospital setting; presence and type of manifestation of potential rheumatologic interest, at the onset of disease or during its evolution as overall prevalence.

Quality of the included studies was assessed using the Cochrane risk of bias tool [13] for randomized trials, the Newcastle-Ottawa scale [14] for non-randomized studies, and the Johanna-Briggs Institute critical appraisal tools [15] for case reports and case series.

### Statistical analysis

A meta-analysis was performed to estimate the overall proportion of COVID-19 patients presenting with a clinical manifestation of potential rheumatologic interest. Considering the substantial heterogeneity expected, we adopted a random-effects model to pool data from included studies. Forest plots were used to graphically represent the effect size, which was the pooled prevalence of the clinical manifestation of interest, as initial symptom or during COVID-19 infection, at 95% confidence intervals. *I*^2^ was calculated to measure between-studies heterogeneity. Publication bias was assessed with Egger’s regression test after visual inspection of funnel plot [16]. In case of publication bias, the *“trim and fill”* method was used to re-calculate pooled estimates [17].

## Results

**Fig. 1:**
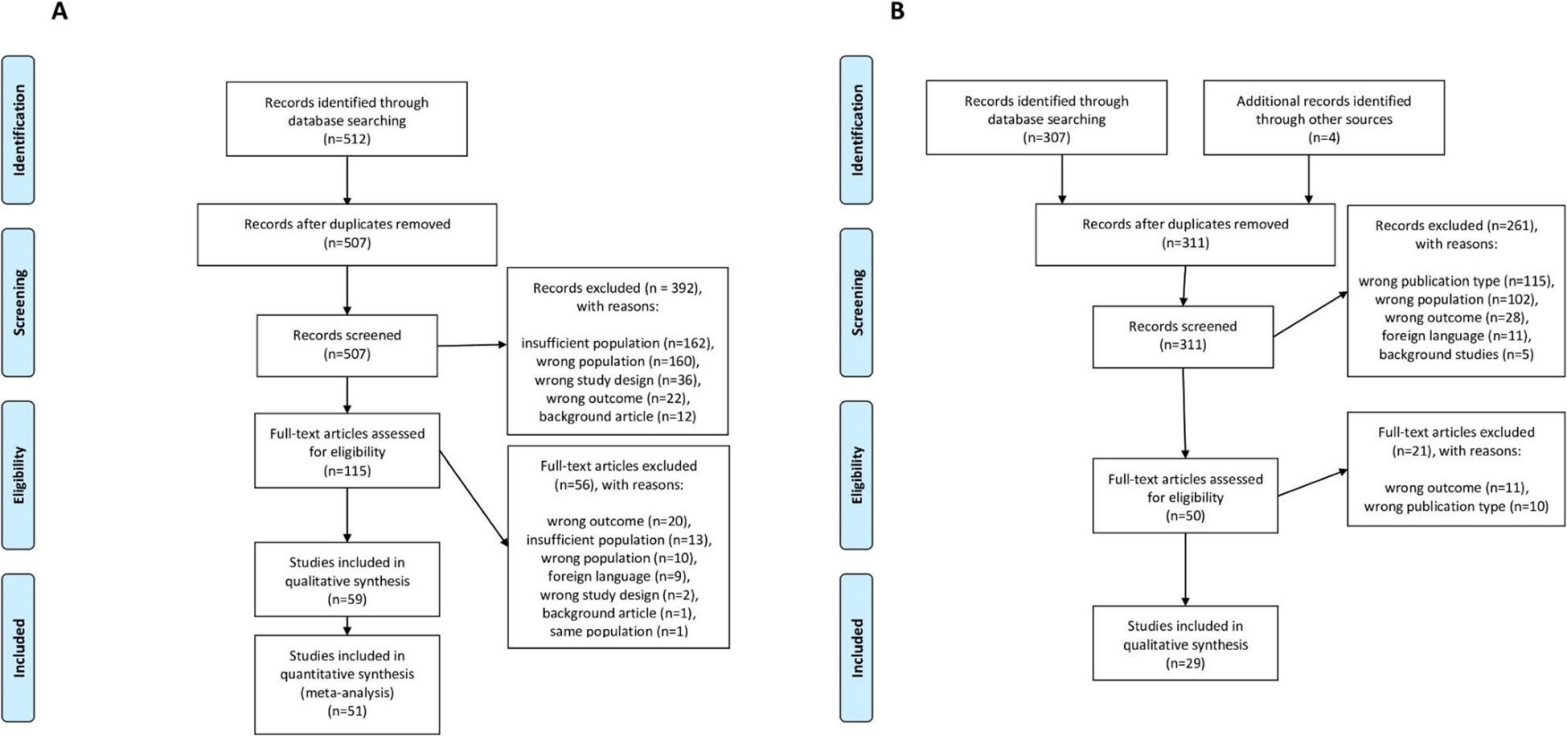
Flowchart of the article selection process from first (A) and second string (B)

The search strategy identified 512 articles from the first string and 307 from the second (Fig. 1a and b). Four additional papers were identified through manual search of references. Respectively from the first and the second search, 115 and 50 studies were considered potentially relevant for full text evaluation. Overall, the full article review identified 88 studies that proceeded to data extraction and analysis. Of these, 51 articles were included in the meta-analysis. Characteristics of the selected studies are shown in Table 1. Three RCTs were included and they were considered at moderate risk of bias (Table S1). Of the 59 articles evaluated through the Newcastle-Ottawa scale, 41 were rated of good quality, 10 of fair quality, and 8 of poor quality (Table S2). Finally, 15 case series and 11 case reports were deemed eligible after the application of Johanna-Briggs critical appraisal tools.

**Table 1:** Characteristics of the included studies

Study	Year	Journal	Country	Study design	Total patients	Setting	Findings of rheumatologic interest
Alramthan	2020	Clin Exp Dermatol	Kuwait	case report	2	non-hospital	acro-ischemic lesions
Andina	2020	Pediatr Dermatol	Spain	case series	22	non-hospital	chilblains
Beyrouty	2020	J Neurol Neurosurg Psychiatry	UK	case series	6	hospital	antiphospholipid antibodies
Bomhof	2020	Br J Haematol	Netherlands	case series	3	hospital	immune thrombocytopenia
Bouaziz	2020	J Eur Acad Dermatol Venereol	France	case series	7	non-hospital	chilblains
Bowles	2020	NEJM	UK	case series	35	hospital	antiphospholipid antibodies
Cao	2020	Clin Infect Dis	China	retrospective observational study	102	hospital	myalgia (prevalence); fatigue (prevalence)
Castelnovo	2020	J Eur Acad Dermatol Venereol	Italy	case series	2	non-hospital	cutaneous vasculitis
Chen	2020	J Infect	China	retrospective observational study	249	hospital	fatigue (onset)
Chen	2020	Acta Ophthalmol	China	retrospective observational study	535	non-hospital	fatigue (prevalence); arthralgia or myalgia (prevalence)
Chen	2020	Infection	China	retrospective observational study	145	hospital	myalgia (prevalence); fatigue (prevalence)
Chen	2020	Chest	China	retrospective observational study	1590	hospital	fatigue (prevalence on 1365 patients); myalgia or arthralgia (prevalence on 1338 patients)
Chen	2020	J Gerontol a Bio Sci Med Sci	China	retrospective observational study	203	hospital	fatigue (prevalence); myalgia or arthralgia (prevalence)
Chen	2020	BMJ	China	retrospective observational study	274	hospital	myalgia (onset); fatigue (onset)
Chen	2020	Diabetes Care	China	retrospective observational study	904	hospital	myalgia (onset); fatigue (onset)
Colonna	2020	Pediatr Dermatol	Italy	case series	4	non-hospital	chilblains
Cordoro	2020	Pediatr Dermatol	USA	case series	6	non-hospital	chilblains
de Masson	2020	J Am Acad Dermatol		retrospective observational study	277	non-hospital	chilblains
Dogan	2020	Brain Behav Immun	Turkey	case series	6	hospital	autoimmune encephalitis
Du	2020	Ann Am Thorac Soc	China	retrospective observational study	109	hospital	myalgia (onset); fatigue (onset)
Galeano-Valle	2020	Thromb Res	Spain	retrospective observational study	24	hospital	antiphospholipid antibodies
Galván-Casas	2020	Br J Dermatol	Spain	retrospective observational study	375	hospital and non-hospital	pseudo-chillblain (prevalence); livedo/necrosis: (prevalence)
Garazzino	2020	Euro Surveill	Italy	retrospective observational study	168 children	hospital and non-hospital	fatigue (prevalence)
Harzallah	2020	J Thromb Haem	France	case series	56	not-reported	antiphospholipid antibodies
Hu	2020	Phytomedicine	China	RCT	284	hospital	fatigue (prevalence)
Huang	2020	J Med Virol	China	retrospective observational study	344	hospital	fatigue (prevalence)
Huang	2020	PLOS Negl Trop Dis	China	retrospective observational study	202	hospital	myalgia (onset); fatigue (onset)
Hung	2020	Lancet	Hong Kong	RCT	127	hospital	myalgia (prevalence); malaise (prevalence)
Hur	2020	Otolaryngol Head Neck Surg	USA	retrospective observational study	486	hospital	fatigue (prevalence)
Javanian	2020	Rom J Int Med	Iran	retrospective observational study	100	hospital	myalgia (prevalence); fatigue (prevalence)
Ji	2020	Epidemiol Infect	China	retrospective observational study	101	hospital	myalgia (prevalence); fatigue (prevalence)
Jones	2020	Hosp Pediatr	USA	case report	1	hospital	Kawasaki disease
Klopfenstein	2020	Clin Res Hepato	France	retrospective observational study	114	hospital and non-hospital	fatigue (prevalence)
Kolivras	2020	JAAD Case Rep	USA	case report	1	non-hospital	chilblains
Lazarian	2020	Br J Haematol	France	case series	7	hospital	autoimmune hemolytic anemia
Li	2020	Clin Infect Dis	China	retrospective observational study	105	hospital	fatigue (prevalence)
Li	2020	Br J Haematol	USA	case report	1	hospital	Evans syndrome
Lian	2020	Clin Infect Dis	China	retrospective observational study	788	hospital	myalgia (prevalence); fatigue (prevalence)
Liguori	2020	Brain Behav Im	Italy	retrospective observational study	103	hospital	myalgia (prevalence); fatigue (prevalence)
Liu	2020	J Clin Virol	China	retrospective observational study	140	hospital	myalgia (onset); fatigue (onset)
Liu	2020	J Infect	China	retrospective observational study	245	hospital	myalgia (prevalence); fatigue (prevalence)
Lopez	2020	Br J Haematol	USA	case report	1	hospital	autoimmune hemolytic anemia
Lu	2020	Pediatr Infect	China	retrospective observational study	110 children	hospital	fatigue (prevalence)
Meng	2020	Plos Pathog	China	retrospective observational study	168	hospital	myalgia (prevalence); fatigue (prevalence)
Menter	2020	Histopathology	Switzerland	case series	21	post-mortem	vasculitis of the pulmonary veins and capillaries
Mo	2020	Clin Infect Dis	China	retrospective observational study	155	hospital	fatigue (prevalence); myalgia or arthralgia (prevalence)
Moeinzadeh	2020	Iran J Kidney Dis	Iran	case report	1	hospital	glomerulonephritis and ANCA positivity
Nowak	2020	Pol Arch Intern	Poland	retrospective observational study	169	hospital	fatigue (prevalence)
Paderno	2020	Int Forum Allergy Rhinol	Italy	retrospective observational study	295	hospital and non-hospital	arthromyalgia (prevalence and onset)
Palaiodimos	2020	Metabolism	USA	retrospective observational study	200	hospital	myalgia (onset)
Pan	2020	Am J Gastroenterol	China	retrospective observational study	103	hospital	myalgia (prevalence)
Pilotto	2020	Ann Neurol	Italy	case report	1	hospital	autoimmune encephalitis
Qi	2020	Int J Infect Dis	China	retrospective observational study	147	hospital	fatigue (prevalence)
Redd	2020	Gastroenterology	USA	retrospective observational study	318	hospital	myalgia (onset); fatigue (onset); arthralgia (onset)
Ren	2020	Intensive Care	China	retrospective observational study	150	hospital	myalgia (prevalence); fatigue (prevalence)
Rivera-Figueroa	2020	Indian Pediatr	India	case report	1	hospital	Kawasaki disease
Sedaghat	2020	J Clin Neurosci	Iran	case report	1	hospital	Guillain Barre syndrome
Shi	2020	Diabetes Care	China	retrospective observational study	306	hospital	myalgia (prevalence); fatigue (prevalence)
Shi	2020	JAMA Cardiol	China	retrospective observational study	416	hospital	myalgia (prevalence); fatigue (prevalence)
Suarez-Valle	2020	J Eur Acad Dermatol Venereol	Spain	case series	3	hospital	acro-ischemic lesions
Tang	2020	BMJ	China	RCT	150	hospital	fatigue (prevalence on 136 pts)
Tian	2020	J infect	China	retrospective observational study	262	hospital	fatigue (prevalence)
Vanegas-Ramirez	2020	J Eur Acad Dermatol Venereol	Germany	case report	1	hospital	vasculitis
Verdoni	2020	Lancet	Italy	retrospective observational study	10	hospital	Kawasaki disease
Wang	2020	JAMA	China	retrospective observational study	138	hospital	myalgia (prevalence); fatigue (prevalence)
Wang	2020	Crit Care	China	retrospective observational study	107	hospital	myalgia (prevalence); fatigue (prevalence)
Wang	2020	J Med Virol	China	retrospective observational study	889	non-hospital	myalgia (prevalence); fatigue (prevalence)
Wang	2020	Int J Inf Dis	China	retrospective observational study	125	hospital	myalgia (prevalence); fatigue (prevalence)
Wang	2020	Diabetes Res Clin	China	retrospective observational study	132	hospital	myalgia (prevalence); fatigue (prevalence)
Wu	2020	JAMA Intern Med	China	retrospective observational study	201	hospital	myalgia or fatigue (onset)
Yan	2020	BMJ open diabetes res care	China	retrospective observational study	193	hospital	fatigue (prevalence)
Yang	2020	J Infect	China	retrospective observational study	149	hospital	myalgia (prevalence)
Yao	2020	Pol Arch Intern	China	retrospective observational study	108	hospital	myalgia or fatigue (prevalence)
Zhang	2020	J Infect Dis	China	retrospective observational study	112	hospital	myalgia (prevalence); fatigue (prevalence)
Zhang	2020	Diabetes Obes Metab	China	retrospective observational study	166	hospital	myalgia (prevalence); fatigue (prevalence)
Zhang	2020	NEJM	China	case series	3	hospital	antiphospholipid antibodies
Zhang	2020	J Clin Virol	China	retrospective observational study	221	hospital	fatigue (prevalence)
Zhang	2020	J Clin Virol	China	retrospective observational study	111	hospital	myalgia (prevalence); fatigue (prevalence)
Zhang	2020	Eur Radiol	China	retrospective observational study	120	hospital	myalgia or fatigue (onset)
Zhao	2020	AJR Am J Roentgerol	China	retrospective observational study	101	hospital	myalgia or fatigue (onset)
Zheng	2020	Eur Rev. Med Pharmacol Sci	China	retrospective observational study	161	hospital	myalgia (prevalence); fatigue (prevalence)
Zheng	2020	Clin Chem Lab Med	China	retrospective observational study	141	hospital	fatigue (prevalence)
Zhou	2020	Lancet	China	retrospective observational study	191	hospital	myalgia (prevalence); fatigue (prevalence)
Zhou	2020	Eur Radiol	China	retrospective observational study	100	hospital	myalgia (prevalence); fatigue (prevalence)
Zhou	2020	Clin Exp Hypert	China	retrospective observational study	110	hospital	myalgia (prevalence); fatigue (prevalence)
Zhou	2020	Plos One	China	retrospective observational study	366	hospital	fatigue (prevalence); myalgia and arthralgia (prevalence)
Zhou	2020	Clin Trans Sci	China	case series	6	hospital	ANA and ENA
Zulfiqar	2020	NEJM	France	case report	1	hospital	immune thrombocytopenic purpura

### Arthralgia in COVID-19

Redd et al. [18] reported a proportion of arthralgia at presentation of 2.5% in 318 COVID-19 patients, but this was the only study describing the prevalence of arthralgia as independent symptom. Six additional articles reported the presence of arthralgia, but always combined with myalgia. Therefore, we deemed the symptom “arthralgia” not suitable for meta-analysis.

Paderno et al. [19] showed that 9.4% of COVID-19 patients complained of arthromyalgia at disease onset, while the overall prevalence was 50.4%. Zhou et al. [20] reported that only 3.8% of 366 hospitalized patients had myalgia or arthralgia, while Chen et al. [21] described a proportion of 17.5% in 1338 patients. In a cohort of 203 hospitalized patients, Chen and colleagues [22] observed that myalgia or arthralgia were present in 26.6% of cases and, describing the characteristics of 535 hospitalized and non-hospitalized COVID-19 patients, Chen et al. [23] found arthralgia or myalgia in 29% of cases, whereas Mo et al. [24] reported a prevalence of 61%.

### Myalgia in COVID-19

Thirty-three articles describing the proportion of COVID-19 patients experiencing myalgia were included in the meta-analysis. Of these, 7 explored myalgia at disease onset [18, 25–30] and 26 during COVID-19 evolution [31–56].

**Fig. 2:**
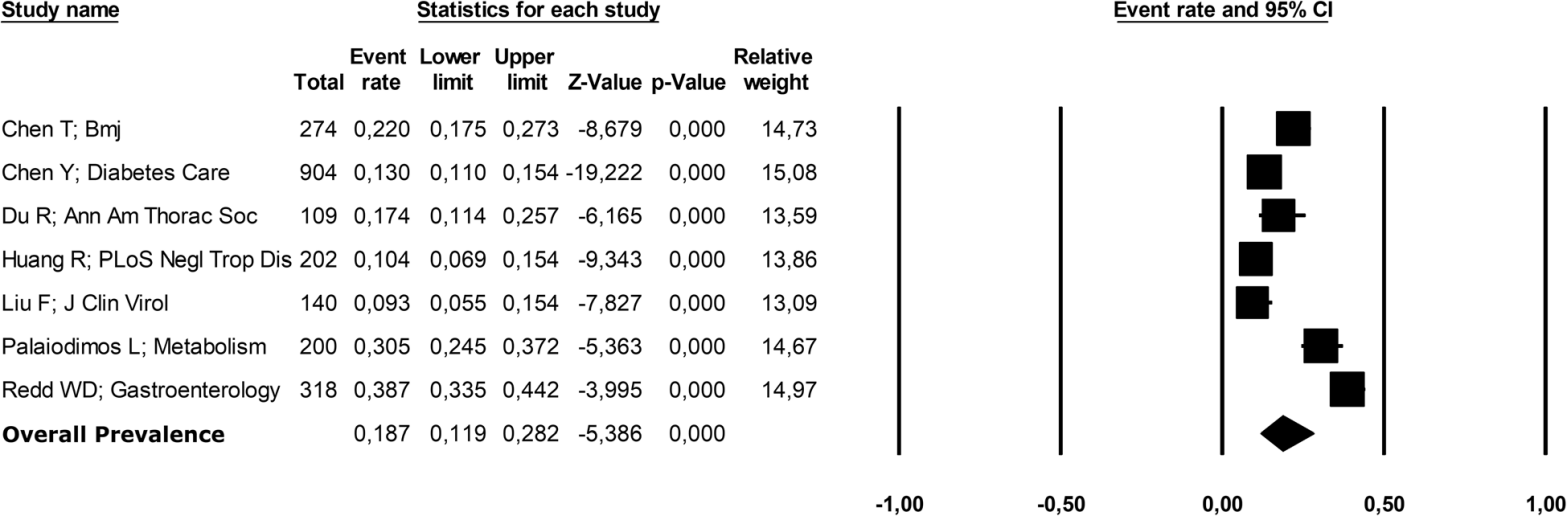
Meta-analysis of muscle pain prevalence as initial symptom of COVID-19

Pooled estimate of muscle pain as initial symptom was 0.187 (95% CI 0.119–0.282, *p* < 0.001) (Fig. 2). The *I*^*2*^ was calculated to be 95.2%. Visual inspection of funnel plot (Fig. S1) suggested potential publication bias. Hence, we applied the *“trim and fill”* method to adjust funnel plot asymmetry and correct the variance. Two studies were removed and, after the imputed fills were added, the adjusted point estimate remained unchanged.

**Fig. 3:**
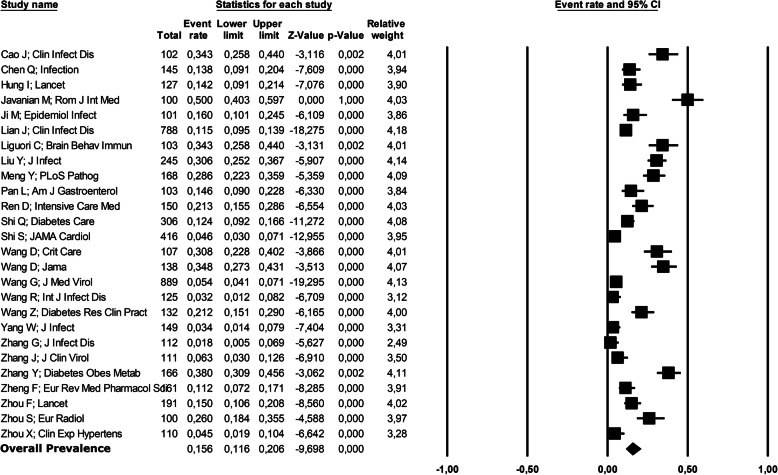
Meta-analysis of muscle pain prevalence during the course of COVID-19

Pooled estimate of the prevalence of muscle pain in patients with COVID-19 was 0.156 (95% CI 0.116–0.206) (Fig. 3). The *I*^*2*^ was calculated to be 94.3%. Funnel plot is shown in Fig. S2. The *“trim and fill”* procedure excluded two studies and, filling the missing effect sizes, resulted in an adjusted value of 0.172 (95 CI 0.129–0.226).

Moreover, 4 additional papers reported the proportion of patients experiencing myalgia combined with fatigue. At disease onset the prevalence of both symptoms aggregated varied between 16.9% [57], 32.3% [58], and 48% [59]. Furthermore, Yao et al. [60] described a prevalence of 25.9% during the course of the illness.

### Fatigue in COVID-19

Forty-seven articles describing the percentage of COVID-19 patients complaining of fatigue were included in the meta-analysis. Of these, 7 explored fatigue at disease onset [18, 25–29, 61] and 40 during its evolution [20–24, 31, 32, 34–39, 41–48, 50–56, 62–73].

**Fig. 4:**
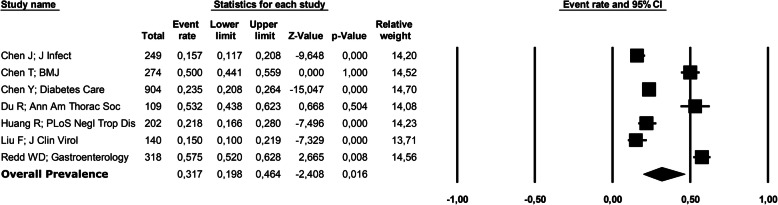
Meta-analysis of fatigue prevalence as initial symptom of COVID-19

**Fig. 5:**
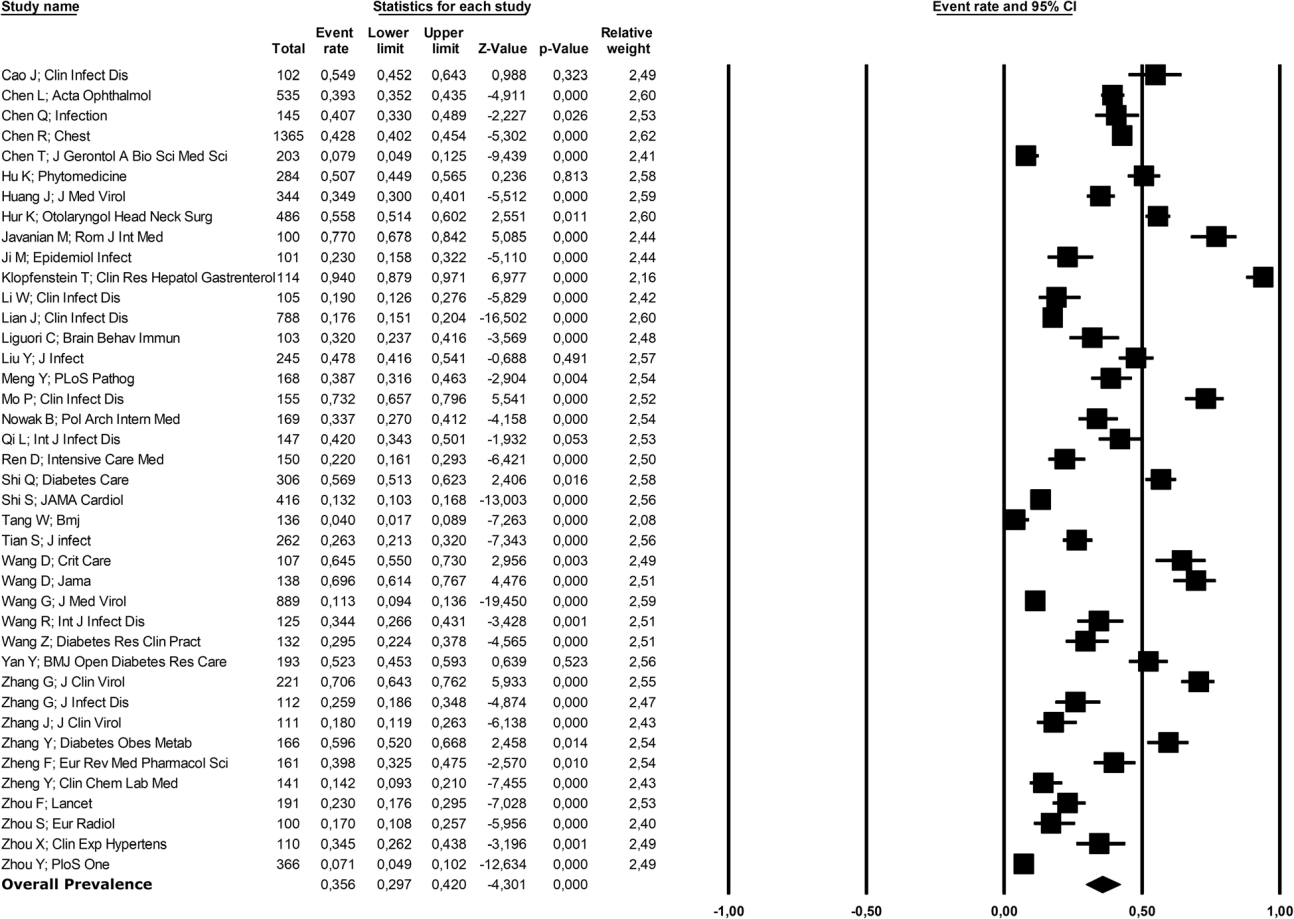
Meta-analysis of fatigue prevalence during the course of COVID-19

Pooled estimate of fatigue as initial symptom was 0.317 (95% CI 0.198–0.464) (Fig. 4). An *I*^*2*^ of 97.4% was calculated. Visual inspection of funnel plot (Fig. S3) suggested no potential publication bias. Pooled estimate of prevalence of fatigue in patients with COVID-19 was 0.356 (95% CI 0.297–0.420) (Fig. 5). The *I*^*2*^ was 97.1%. No plot (Fig. S4) asymmetry was detected.

Additionally, Garazzino et al. [74] and Lu et al. [75] reported prevalence of fatigue respectively of 1.8 and 3.6% in children affected by COVID-19. These latter studies were not included in the meta-analysis as the only ones not describing data of an adult population.

### Vasculitis in COVID-19

We found 7 articles reporting the occurrence of vasculitis in COVID-19 patients. With the exception of the study by Menter et al. [76], all articles were case series or case reports describing only patients with vasculitis. Verdoni et al. [77] described an increased incidence of Kawasaki disease, a medium-sized-vessel vasculitis, after the appearance of SARS-CoV-2 in the Italian province of Bergamo. The authors reported data about 10 recently diagnosed patients (age range 2.9 to 16 years, mean age 7.5 ± 3.5 years) with Kawasaki vasculitis, 80% of them also having positive serology for SARS-CoV-2, and presenting with clinical and biochemical characteristics different from those observed in the historical Kawasaki cohort of that region. Other two cases of Kawasaki disease in paediatric age related to COVID-19 were shown by Rivera-Figueroa et al. [78] and by Jones et al. [79], while Menter et al. [76] described post-mortem findings of 21 adult COVID-19 patients revealing, in one case, florid vasculitis of pulmonary veins and capillaries. Moreover, Vanegas-Ramirez et al. [80] observed a skin rash and vasculitis in a 57-year-old COVID-19 patient, Castelnovo et al. [81] discussed 2 cases of cutaneous vasculitis in young COVID-19 patients, and Moeinzadeh et al. [82] reported the case of a 25-year-old COVID-19 patient presenting with glomerulonephritis and positive test for anti-neutrophilic cytoplasmic antibodies (ANCA).

### Chilblains in COVID-19

We included 9 articles reporting the presence of chilblains in COVID-19. Galván-Casas et al. [83] presented data about the classification of lesions in acral areas of 375 COVID-19 patients, outlining a prevalence of 19% for the pseudo-chilblain pattern. Investigating 277 patients during the COVID-19 outbreak, De Masson et al. [84] described the presence of 106 chilblain lesions, although only 9% of patients had a positive test for SARS-CoV-2. Similarly, Kolivras et al. [85] observed, in a case report, a 23-year-old man of chilblains induced by COVID-19 and Cordoro et al. [86] reported a case series of 6 paediatric age patients with chilblains as cutaneous reaction to SARS-CoV-2. Colonna et al. [87] described in a case series the finding of chilblains in 4 children suspected for COVID-19, while Bouaziz et al. [88] reported chilblains in 2 of the 14 studied COVID-19 patients and Andina et al. [89] described the case series of 22 children presenting with chilblains during the COVID outbreak, although only one tested positive. Finally, Suarez-Valle et al. [90] and Alramthan et al. [91] described respectively 3 and 2 COVID-19 patients with acro-ischemic lesions.

### Autoantibodies in COVID-19

We found 6 articles describing the presence of autoantibodies in patients with COVID-19. Zhou et al. [92] found a prevalence of 20% for anti-52 kDa SSA/Ro antibody, of 25% for anti-60 kDa SSA/Ro antibody and of 50% for antinuclear antibody in their cases. Zhang et al. [93] described a case series of three patients with positive antiphospholipid antibodies (aPL), and in particular anticardiolipin IgA, anti-β2-glycoprotein I IgA and IgG. Out of 24 cases, Galeano-Valle et al. [94], studying 24 patients hospitalized with COVID-19 and venous thromboembolism, found 2 patients weakly positive for anticardiolipin IgM and anti–β2-glycoprotein I IgM, while anticardiolipin IgG and anti–β2-glycoprotein I IgG were negative in all patients. Harzallah et al. [95] studied 56 COVID-19 cases showing that 45% were lupus anticoagulant (LAC) positive whereas in 10% anticardiolipin or anti–β2-glycoprotein I IgG and IgM were detected. Similarly, Bowles et al. [96] found a positive LAC in 91% of the 35 studied COVID-19 patients with prolonged activated partial-thromboplastin time (aPTT). Moreover, in a small case series, Beyrouti et al. [97] reported that 5 of 6 patients had a positive LAC, one with medium-titre IgM anticardiolipin and low-titre IgG and IgM anti-β2-glycoprotein1 antibodies.

### Haematological manifestations of COVID-19 of potential rheumatologic interest

We retrieved 5 articles assessing haematological manifestation of COVID-19 of potential rheumatologic interest. All articles were case series or case reports describing only patients with the specific haematological conditions. Lazarian et al. [98] and Lopez et al. [99] showed the occurrence of autoimmune haemolytic anaemia respectively in 7 and 1 patients affected by COVID-19. Bomhof et al. [100] found three cases of immune-mediated thrombocytopenia related to COVID-19 while Zulfiqar et at [101]. observed the occurrence of immune thrombocytopenic purpura in a patient with COVID-19 and Li et al. [102] reported a patient with Evans syndrome, which is characterized by a combination of autoimmune haemolytic anaemia and immune thrombocytopenia.

### Neurological manifestations of COVID-19 of potential rheumatologic interest

Three articles describing neurological manifestations of COVID-19 of potential rheumatologic interest were included. All articles were case series or case reports describing only patients with the specific neurological conditions. Six cases of autoimmune encephalitis were reported by Dogan et al. [103] and an additional case was presented by Pilotto et al. [104]. Moreover, Sedaghat et al. [105] described the case of a COVID-19 patient developing Guillain-Barre syndrome.

## Discussion

We performed a systematic review and meta-analysis with the aim of assessing the occurrence of rheumatic manifestations in patients affected by COVID-19. The recent SARS-CoV-2 pandemic resulted in an exceptional literature contribution; therefore, we were able to include in our review 88 original references, all published as final, peer-reviewed articles, in the last few weeks. Unfortunately, we retrieved only one article describing the prevalence of arthralgia as discrete symptom at disease onset, while 6 additional studies showed the prevalence of arthralgia combined with myalgia, with percentages ranging from 3.8% [20] to 61% [24].

Our meta-analysis shows that muscle pain and fatigue are present respectively in 19 and 32% of patients as initial presentation of COVID-19, while the overall prevalence estimates are 16 and 36% throughout the course of the illness. Moreover, we found additional studies focusing on less common musculoskeletal or autoimmune manifestations of COVID-19, of potential interest for the rheumatologist. Vasculitis, chilblains, presence of autoantibodies commonly found in patients with rheumatic diseases, or autoimmune haematological and neurological disorders have all been reported in patients with COVID-19, although evidence from large cohort studies is still lacking.

Focusing the attention on the only two items we were able to meta-analyse in our review, it is crucial to point out how muscle pain and fatigue are among the most frequent complains in patients with rheumatic diseases. For instance, muscle pain is reported in 16% of patients with rheumatoid arthritis [106] and in up to 100% of polymyalgia rheumatica cases [107], not necessarily accompanied by stiffness [106]. Similarly, 30–35% of patients with dermatomyositis/polymyositis [108, 109] have myalgia, but the proportion rises to 74% in newly diagnosed cases [109]. Comparable figures were observed by Noda et al. [110], with 71% of patients with dermatomyositis and 25% with polymyalgia rheumatica complaining of muscle pain. However, myalgia is a highly-reported symptom even in other connective tissue diseases. In systemic sclerosis, the frequency of muscle pain varies from 20 to 86% [111, 112] and it is between 40 and 80% in systemic lupus erythematosus [113]. In vasculitis, 48% of patients with microscopic polyangitis have been reported to complain of muscle pain [114], while different cases of myalgia as initial symptom of polyarteritis nodosa [115] or of ANCA-associated vasculitis [116] have been described. Moreover, malaise or fatigue are reported in about 30% of patients with polymyalgia rheumatica [107] and 42–69% of people with rheumatoid arthritis [117]. In an international survey including over 6000 participants [118], patients with rheumatoid arthritis, systemic lupus erythematosus, ankylosing spondylitis, Sjögren’s syndrome, psoriatic arthritis, or systemic sclerosis, presented severe fatigue in 41 to 57% of cases. Even higher figures were observed in systemic sclerosis by Richards et al., with 75% of patients complaining of fatigue [119], or in systemic lupus erythematosus, where fatigue was identified as one of the primary symptoms in 53–80% of patients [120]. Similarly, in ANCA-associated vasculitis, fatigue is considered a common symptom, reported in 75% of patients [121]. Finally, Grayson et al. assessed fatigue through a dedicated scale, the “multidimensional fatigue inventory 20”, in 692 patients with 9 different forms of systemic vasculitis, observing that 76% of cases had a score > 13, indicative of severe fatigue [122].

Arthralgia, myalgia and fatigue are the most common symptoms leading to referral of patients to a rheumatologist. As outlined by our systematic review and meta-analysis, 19% of COVID-19 cases might present muscle pain as initial symptom, while 32% might present fatigue. It is therefore conceivable that, especially for individuals with non-specific or mild complaints and without respiratory distress, a proportion of COVID-19 patients might be referred to the rheumatologist early in the disease course. Rheumatologists should hereafter bear in mind COVID-19 as a possible differential diagnosis.

However, some limitations must be considered. Although we were able to include a considerable number of studies in our review, knowledge about COVID-19 is a rapidly evolving process and the global pandemic represents a constantly expanding field of research, with new data contributed daily. As such, our work provides preliminary information, that will need to be implemented and confirmed by forthcoming research. In this view, future studies with longitudinal follow-up of COVID-19 patients would provide useful data for a research agenda which, in our opinion, should address the following issues: (a) geographical differences in prevalence and characteristics of COVID-19 manifestations of potential rheumatologic interest; (b) clinical persistence and evolution of symptoms as arthralgia, myalgia and fatigue after resolution of the acute infection; (c) need for long-term follow-up and, where appropriate, treatment of COVID-19 manifestations of potential rheumatologic interest; (d) monitoring patients for possible late-onset post-infective complications of potential rheumatologic interest.

A second limitation of our study is for instance the geographic origin of the included literature. The majority of retrieved articles were contributed from a single country, China, consistently with the first identification site of the novel coronavirus and the consequent interest of the Chinese scientific community towards the outbreak, but representing a source of bias and preventing the possibility to confidently generalize our findings to other populations, particularly of non-Asian ancestry. Besides that, evidence from non-hospitalized patients was limited and the clinical characteristics of cases with different disease severity were not homogeneously reported. As a result, we could not perform meta-regression or subgroup analysis to evaluate the effect of different setting of COVID-19 care or the peculiarities between critically-ill and mildly diseased patients. Finally, arthralgia as a unique symptom was poorly represented and we could not meta-analyse it.

## Conclusions

In conclusion, our systematic review and meta-analysis suggests that symptoms of potential rheumatologic interest are frequently reported in COVID-19, both at onset or throughout the disease course. Accordingly, as implication for clinical practice, we would raise the awareness on the possibility that the new global threat might show up in the rheumatology office.

## Supplementary information

**Additional file 1 Table S1.** Quality assessment of randomized clinical trials. **Table S2.** Quality assessment of observational studies. **Figure S1.** Funnel plot. Meta-analysis of muscle pain as presenting symptom of COVID-19. **Figure S2.** Funnel plot. Meta-analysis of muscle pain prevalence during the course of COVID-19. **Figure S3.** Funnel plot. Meta-analysis of fatigue as presenting symptom of COVID-19. **Figure S4.** Funnel plot. Meta-analysis of fatigue prevalence during the course of COVID-19.

## Data Availability

All relevant data are reported within the manuscript or in the cited references.

## Abbreviations

SARS-CoV-2: Severe acute respiratory virus 2
COVID-19: Coronavirus disease 2019
SARS: Severe acute respiratory syndrome SARS
MERS: Middle East respiratory syndrome
ACE2: Angiotensin-converting enzyme 2
RCTs: Randomised controlled trials
PRISMA: Preferred Reporting Items for Systematic Reviews and Meta-Analyses
ANCA: Anti-neutrophilic cytoplasmic antibodies
aPL: Antiphospholipid antibodies
LAC: Lupus anticoagulant
aPTT: Activated partial-thromboplastin time

## References

[1] Phelan AL, Katz R, Gostin LO. The Novel Coronavirus Originating in Wuhan, China: Challenges for Global Health Governance. JAMA. 2020;10.1001/jama.2020.1097. 10.1001/jama.2020.1097. 10.1001/jama.2020.1097 31999307

[2] Peiris JS, Yuen KY, Osterhaus AD, Stöhr K (2003). The severe acute respiratory syndrome. N Engl J Med.

[3] Zumla A, Hui DS, Perlman S (2015). Middle East respiratory syndrome. Lancet..

[4] Jiang F, Deng L, Zhang L, Cai Y, Cheung CW, Xia Z (2020). Review of the clinical characteristics of coronavirus disease 2019 (COVID-19). J Gen Intern Med.

[5] Verdecchia P, Cavallini C, Spanevello A, Angeli F (2020). The pivotal link between ACE2 deficiency and SARS-CoV-2 infection. Eur J Intern Med.

[6] Li MY, Li L, Zhang Y, Wang XS (2020). Expression of the SARS-CoV-2 cell receptor gene ACE2 in a wide variety of human tissues. Infect Dis Poverty.

[7] Marks M, Marks JL (2016). Viral arthritis. Clin Med (Lond).

[8] Hoang MP, Park J, Hoang MP, Selim MA (2020). Vasculitis. Hospital-based Dermatopathology: an illustrated diagnostic guide.

[9] Moroncini G, Mori S, Tonnini C, Gabrielli A (2013). Role of viral infections in the etiopathogenesis of systemic sclerosis. Clin Exp Rheumatol.

[10] Salvarani C, Cantini F, Boiardi L, Hunder GG (2002). Polymyalgia rheumatica and giant-cell arteritis. N Engl J Med.

[11] Moher D, Liberati A, Tetzlaff J, Altman DG (2009). Preferred reporting items for systematic reviews and meta-analyses: the PRISMA statement. PLoS Med.

[12] Schardt C, Adams MB, Owens T, Keitz S, Fontelo P (2007). Utilization of the PICO framework to improve searching PubMed for clinical questions. BMC Med Inform Decis Mak.

[13] Higgins JP, Altman DG, Gotzsche PC, Juni P, Moher D, Oxman AD (2011). The Cochrane Collaboration's tool for assessing risk of bias in randomised trials. Bmj..

[14] Wells GASB, O’Connell D, Peterson J, Welch V, Losos M (2019). The Newcastle-Ottawa scale (NOS) for assessing the quality of nonrandomised studies in meta-analysis.

[15] Johanna-Briggs I (2017). Critical appraisal tools.

[16] Lin L, Chu H (2018). Quantifying publication bias in meta-analysis. Biometrics..

[17] Duval S, Tweedie R (2000). Trim and fill: a simple funnel-plot-based method of testing and adjusting for publication bias in meta-analysis. Biometrics..

[18] Redd WD, Zhou JC, Hathorn KE, et al. Prevalence and Characteristics of Gastrointestinal Symptoms in Patients With Severe Acute Respiratory Syndrome Coronavirus 2 Infection in the United States: a Multicenter Cohort Study. Gastroenterology. 2020;159(2):765–767.e2. 10.1053/j.gastro.2020.04.045. 10.1053/j.gastro.2020.04.045 PMC7195377 32333911

[19] Paderno A, Schreiber A, Grammatica A, et al. Smell and taste alterations in COVID-19: a cross-sectional analysis of different cohorts. Int Forum Allergy Rhinol. 2020;10(8):955–62. 10.1002/alr.22610. 10.1002/alr.22610 PMC7272886 32410386

[20] Zhou Y, He Y, Yang H, Yu H, Wang T, Chen Z (2020). Development and validation a nomogram for predicting the risk of severe COVID-19: a multi-center study in Sichuan, China. PLoS One.

[21] Chen R, Liang W, Jiang M, et al. Risk Factors of Fatal Outcome in Hospitalized Subjects With Coronavirus Disease 2019 From a Nationwide Analysis in China. Chest. 2020;158(1):97–105. 10.1016/j.chest.2020.04.010. 10.1016/j.chest.2020.04.010 PMC7158802 32304772

[22] Chen T, Dai Z, Mo P, et al. Clinical characteristics and outcomes of older patients with coronavirus disease 2019 (COVID-19) in Wuhan, China (2019): a single-centered, retrospective study. J Gerontol A Biol Sci Med Sci. 2020;glaa089. 10.1093/gerona/glaa089. 10.1093/gerona/glaa089 PMC7184388 32279081

[23] Chen L, Deng C, Chen X, et al. Ocular manifestations and clinical characteristics of 535 cases of COVID-19 in Wuhan, China: a cross-sectional study. Acta Ophthalmol. 2020;10.1111/aos.14472. 10.1111/aos.14472. 10.1111/aos.14472 PMC7276826 32421258

[24] Mo P, Xing Y, Xiao Y, et al. Clinical characteristics of refractory COVID-19 pneumonia in Wuhan, China. Clin Infect Dis. 2020;ciaa270. 10.1093/cid/ciaa270.

[25] Chen T, Wu D, Chen H, Yan W, Yang D, Chen G (2020). Clinical characteristics of 113 deceased patients with coronavirus disease 2019: retrospective study. Bmj..

[26] Chen Y, Yang D, Cheng B, et al. Clinical Characteristics and Outcomes of Patients With Diabetes and COVID-19 in Association With Glucose-Lowering Medication. Diabetes Care. 2020;43(7):1399–407. 10.2337/dc20-0660. 10.2337/dc20-0660 32409498

[27] Du RH, Liu LM, Yin W, et al. Hospitalization and Critical Care of 109 Decedents with COVID-19 Pneumonia in Wuhan, China. Ann Am Thorac Soc. 2020;17(7):839–46. 10.1513/AnnalsATS.202003-225OC. 10.1513/AnnalsATS.202003-225OC PMC7328178 32255382

[28] Huang R, Zhu L, Xue L, Liu L, Yan X, Wang J (2020). Clinical findings of patients with coronavirus disease 2019 in Jiangsu province, China: a retrospective, multi-center study. PLoS Negl Trop Dis.

[29] Liu F, Li L, Xu M, Wu J, Luo D, Zhu Y (2020). Prognostic value of interleukin-6, C-reactive protein, and procalcitonin in patients with COVID-19. J Clin Virol.

[30] Palaiodimos L, Kokkinidis DG, Li W, et al. Severe obesity, increasing age and male sex are independently associated with worse in-hospital outcomes, and higher in-hospital mortality, in a cohort of patients with COVID-19 in the Bronx, New York. Metabolism. 2020;108:154262. 10.1016/j.metabol.2020.154262 PMC7228874 32422233

[31] Cao J, Tu WJ, Cheng W, et al. Clinical Features and Short-term Outcomes of 102 Patients with Coronavirus Disease 2019 in Wuhan, China. Clin Infect Dis. 2020;71(15):748–55. 10.1093/cid/ciaa243. 10.1093/cid/ciaa243 PMC7184479 32239127

[32] Chen Q, Zheng Z, Zhang C, et al. Clinical characteristics of 145 patients with corona virus disease 2019 (COVID-19) in Taizhou, Zhejiang, China. Infection. 2020;48(4):543–51. 10.1007/s15010-020-01432-5. 10.1007/s15010-020-01432-5 PMC7186187 32342479

[33] Hung IF, Lung KC, Tso EY, et al. Triple combination of interferon beta-1b, lopinavir-ritonavir, and ribavirin in the treatment of patients admitted to hospital with COVID-19: an open-label, randomised, phase 2 trial. Lancet. 2020;395(10238):1695–704. 10.1016/S0140-6736(20)31042-4. 10.1016/S0140-6736(20)31042-4 PMC7211500 32401715

[34] Javanian M, Bayani M, Shokri M, et al. Clinical and laboratory findings from patients with COVID-19 pneumonia in Babol North of Iran: a retrospective cohort study. Rom J Intern Med. 2020;58(3):161–7. 10.2478/rjim-2020-0013. 10.2478/rjim-2020-0013 32396143

[35] Ji M, Yuan L, Shen W, Lv J, Li Y, Li M (2020). Characteristics of disease progress in patients with coronavirus disease 2019 in Wuhan, China. Epidemiol Infect.

[36] Lian J, Jin X, Hao S, et al. Analysis of Epidemiological and Clinical Features in Older Patients With Coronavirus Disease 2019 (COVID-19) Outside Wuhan. Clin Infect Dis. 2020;71(15):740–7. 10.1093/cid/ciaa242. 10.1093/cid/ciaa242 PMC7184356 32211844

[37] Liguori C, Pierantozzi M, Spanetta M, et al. Subjective neurological symptoms frequently occur in patients with SARS-CoV2 infection. Brain Behav Immun. 2020;88:11–16. 10.1016/j.bbi.2020.05.037. 10.1016/j.bbi.2020.05.037 PMC7235586 32416289

[38] Liu Y, Du X, Chen J, et al. Neutrophil-to-lymphocyte ratio as an independent risk factor for mortality in hospitalized patients with COVID-19. J Infect. 2020;81(1):e6–12. 10.1016/j.jinf.2020.04.002. 10.1016/j.jinf.2020.04.002 PMC7195072 32283162

[39] Meng Y, Wu P, Lu W, Liu K, Ma K, Huang L (2020). Sex-specific clinical characteristics and prognosis of coronavirus disease-19 infection in Wuhan, China: a retrospective study of 168 severe patients. PLoS Pathog.

[40] Pan L, Mu M, Yang P, Sun Y, Wang R, Yan J (2020). Clinical characteristics of COVID-19 patients with digestive symptoms in Hubei, China: a descriptive, cross-sectional, multicenter study. Am J Gastroenterol.

[41] Ren D, Ren C, Yao RQ, Feng YW, Yao YM. Clinical features and development of sepsis in patients infected with SARS-CoV-2: a retrospective analysis of 150 cases outside Wuhan, China. Intensive Care Med. 2020;46(8):1630–3. 10.1007/s00134-020-06084-5. 10.1007/s00134-020-06084-5 PMC7225399 32415313

[42] Shi Q, Zhang X, Jiang F, et al. Clinical Characteristics and Risk Factors for Mortality of COVID-19 Patients With Diabetes in Wuhan, China: a Two-Center, Retrospective Study. Diabetes Care. 2020;43(7):1382–91. 10.2337/dc20-0598. 10.2337/dc20-0598 32409504

[43] Shi S, Qin M, Shen B, et al. Association of Cardiac Injury With Mortality in Hospitalized Patients With COVID-19 in Wuhan, China. JAMA Cardiol. 2020;5(7):802–10. 10.1001/jamacardio.2020.0950. 10.1001/jamacardio.2020.0950 PMC7097841 32211816

[44] Wang D, Hu B, Hu C, Zhu F, Liu X, Zhang J (2020). Clinical characteristics of 138 hospitalized patients with 2019 novel coronavirus-infected pneumonia in Wuhan, China. Jama.

[45] Wang D, Yin Y, Hu C, Liu X, Zhang X, Zhou S (2020). Clinical course and outcome of 107 patients infected with the novel coronavirus, SARS-CoV-2, discharged from two hospitals in Wuhan, China. Crit Care.

[46] Wang G, Chen W, Jin X, Chen YP. Description of COVID-19 cases along with the measures taken on prevention and control in Zhejiang, China. J Med Virol. 2020;10.1002/jmv.25906. 10.1002/jmv.25906. 10.1002/jmv.25906 PMC7264658 32311151

[47] Wang R, Pan M, Zhang X, Han M, Fan X, Zhao F (2020). Epidemiological and clinical features of 125 hospitalized patients with COVID-19 in Fuyang, Anhui, China. Int J Infect Dis.

[48] Wang Z, Du Z, Zhu F (2020). Glycosylated hemoglobin is associated with systemic inflammation, hypercoagulability, and prognosis of COVID-19 patients. Diabetes Res Clin Pract.

[49] Yang W, Cao Q, Qin L, Wang X, Cheng Z, Pan A (2020). Clinical characteristics and imaging manifestations of the 2019 novel coronavirus disease (COVID-19):a multi-center study in Wenzhou city, Zhejiang, China. J Inf Secur.

[50] Zhang G, Nie S, Zhang Z, Zhang Z. Longitudinal Change of Severe Acute Respiratory Syndrome Coronavirus 2 Antibodies in Patients with Coronavirus Disease 2019. J Infect Dis. 2020;222(2):183–8. 10.1093/infdis/jiaa229. 10.1093/infdis/jiaa229 PMC7197530 32358956

[51] Zhang J, Yu M, Tong S, Liu LY, Tang LV (2020). Predictive factors for disease progression in hospitalized patients with coronavirus disease 2019 in Wuhan. China J Clin Virol.

[52] Zhang Y, Li H, Zhang J, et al. The clinical characteristics and outcomes of patients with diabetes and secondary hyperglycaemia with coronavirus disease 2019: a single-centre, retrospective, observational study in Wuhan. Diabetes Obes Metab. 2020;22(8):1443–54. 10.1111/dom.14086. 10.1111/dom.14086 PMC7273002 32406594

[53] Zheng F, Tang W, Li H, Huang YX, Xie YL, Zhou ZG (2020). Clinical characteristics of 161 cases of corona virus disease 2019 (COVID-19) in Changsha. Eur Rev Med Pharmacol Sci.

[54] Zhou F, Yu T, Du R, Fan G, Liu Y, Liu Z (2020). Clinical course and risk factors for mortality of adult inpatients with COVID-19 in Wuhan, China: a retrospective cohort study. Lancet..

[55] Zhou S, Zhu T, Wang Y, Xia L. Imaging features and evolution on CT in 100 COVID-19 pneumonia patients in Wuhan, China. Eur Radiol. 2020;30(10):5446–54. 10.1007/s00330-020-06879-6. 10.1007/s00330-020-06879-6 PMC7197364 32367418

[56] Zhou X, Zhu J, Xu T. Clinical characteristics of coronavirus disease 2019 (COVID-19) patients with hypertension on renin-angiotensin system inhibitors. Clin Exp Hypertens. 2020;42(7):656–60. 10.1080/10641963.2020.1764018. 10.1080/10641963.2020.1764018 PMC7232880 32404011

[57] Zhao W, Zhong Z, Xie X, Yu Q, Liu J (2020). Relation between chest CT findings and clinical conditions of coronavirus disease (COVID-19) pneumonia: a multicenter study. AJR Am J Roentgenol.

[58] Wu C, Chen X, Cai Y, et al. Risk Factors Associated With Acute Respiratory Distress Syndrome and Death in Patients With Coronavirus Disease 2019 Pneumonia in Wuhan, China. JAMA Intern Med. 2020;180(7):1–11. 10.1001/jamainternmed.2020.0994. 10.1001/jamainternmed.2020.0994 PMC7070509 32167524

[59] Zhang R, Ouyang H, Fu L, et al. CT features of SARS-CoV-2 pneumonia according to clinical presentation: a retrospective analysis of 120 consecutive patients from Wuhan city. Eur Radiol. 2020;30(8):4417–26. 10.1007/s00330-020-06854-1. 10.1007/s00330-020-06854-1 PMC7150608 32279115

[60] Yao Q, Wang P, Wang X, et al. A retrospective study of risk factors for severe acute respiratory syndrome coronavirus 2 infections in hospitalized adult patients. Pol Arch Intern Med. 2020;130(5):390–9. 10.20452/pamw.15312. 10.20452/pamw.15312 32329978

[61] Chen J, Qi T, Liu L, Ling Y, Qian Z, Li T (2020). Clinical progression of patients with COVID-19 in Shanghai, China. J Inf Secur.

[62] Hu K, Guan WJ, Bi Y, et al. Efficacy and safety of Lianhuaqingwen capsules, a repurposed Chinese herb, in patients with coronavirus disease 2019: a multicenter, prospective, randomized controlled trial. Phytomedicine. 2020;153242. 10.1016/j.phymed.2020.153242 PMC7229744 33867046

[63] Huang J, Cheng A, Lin S, Zhu Y, Chen G. Individualized prediction nomograms for disease progression in mild COVID-19. J Med Virol. 2020;10.1002/jmv.25969. 10.1002/jmv.25969. 10.1002/jmv.25969 PMC7267495 32369205

[64] Hur K, Price CPE, Gray EL, et al. Factors Associated With Intubation and Prolonged Intubation in Hospitalized Patients With COVID-19. Otolaryngol Head Neck Surg. 2020;163(1):170–8. 10.1177/0194599820929640. 10.1177/0194599820929640 PMC7240317 32423368

[65] Klopfenstein T, Kadiane-Oussou NJ, Royer PY, Toko L, Gendrin V, Zayet S. Diarrhea: An underestimated symptom in Coronavirus disease 2019. Clin Res Hepatol Gastroenterol. 2020;44(3):282–3. 10.1016/j.clinre.2020.04.002. 10.1016/j.clinre.2020.04.002 PMC7183939 32371006

[66] Li W, Zhang B, Lu J, et al. The characteristics of household transmission of COVID-19. Clin Infect Dis. 2020;ciaa450. 10.1093/cid/ciaa450. 10.1093/cid/ciaa450 PMC7184465 32301964

[67] Nowak B, Szymański P, Pańkowski I, et al. Clinical characteristics and short-term outcomes of patients with coronavirus disease 2019: a retrospective single-center experience of a designated hospital in Poland. Pol Arch Intern Med. 2020;130(5):407–11. 10.20452/pamw.15361. 10.20452/pamw.15361 32420710

[68] Qi L, Yang Y, Jiang D, et al. Factors associated with the duration of viral shedding in adults with COVID-19 outside of Wuhan, China: a retrospective cohort study. Int J Infect Dis. 2020;96:531–7. 10.1016/j.ijid.2020.05.045. 10.1016/j.ijid.2020.05.045 PMC7231495 32425636

[69] Tang W, Cao Z, Han M, Wang Z, Chen J, Sun W (2020). Hydroxychloroquine in patients with mainly mild to moderate coronavirus disease 2019: open label, randomised controlled trial. Bmj..

[70] Tian S, Hu N, Lou J, Chen K, Kang X, Xiang Z (2020). Characteristics of COVID-19 infection in Beijing. J Inf Secur.

[71] Yan Y, Yang Y, Wang F, et al. Clinical characteristics and outcomes of patients with severe covid-19 with diabetes. BMJ Open Diabetes Res Care. 2020;8(1):e001343. 10.1136/bmjdrc-2020-001343. 10.1136/bmjdrc-2020-001343 PMC7222577 32345579

[72] Zhang G, Hu C, Luo L, Fang F, Chen Y, Li J (2020). Clinical features and short-term outcomes of 221 patients with COVID-19 in Wuhan, China. J Clin Virol.

[73] Zheng Y, Zhang Y, Chi H, et al. The hemocyte counts as a potential biomarker for predicting disease progression in COVID-19: a retrospective study. Clin Chem Lab Med. 2020;58(7):1106–15. 10.1515/cclm-2020-0377. 10.1515/cclm-2020-0377 32352397

[74] Garazzino S, Montagnani C, Donà D, et al. Multicentre Italian study of SARS-CoV-2 infection in children and adolescents, preliminary data as at 10 April 2020. Euro Surveill. 2020;25(18):2000600. 10.2807/1560-7917.ES.2020.25.18.2000600. 10.2807/1560-7917.ES.2020.25.18.2000600 PMC7219028 32400362

[75] Lu Y, Li Y, Deng W, et al. Symptomatic Infection is Associated with Prolonged Duration of Viral Shedding in Mild Coronavirus Disease 2019: a Retrospective Study of 110 Children in Wuhan. Pediatr Infect Dis J. 2020;39(7):e95–9. 10.1097/INF.0000000000002729. 10.1097/INF.0000000000002729 PMC7279058 32379191

[76] Menter T, Haslbauer JD, Nienhold R, et al. Postmortem examination of COVID-19 patients reveals diffuse alveolar damage with severe capillary congestion and variegated findings in lungs and other organs suggesting vascular dysfunction. Histopathology. 2020;10.1111/his.14134. 10.1111/his.14134. 10.1111/his.14134 PMC7496150 32364264

[77] Verdoni L, Mazza A, Gervasoni A, et al. An outbreak of severe Kawasaki-like disease at the Italian epicentre of the SARS-CoV-2 epidemic: an observational cohort study. Lancet. 2020;395(10239):1771–8. 10.1016/S0140-6736(20)31103-X. 10.1016/S0140-6736(20)31103-X PMC7220177 32410760

[78] Rivera-Figueroa EI, Santos R, Simpson S, Garg P. Incomplete Kawasaki Disease in a Child with Covid-19. Indian Pediatr. 2020;57(7):680–1. 10.1007/s13312-020-1900-0. 10.1007/s13312-020-1900-0 PMC7387257 32393680

[79] Jones VG, Mills M, Suarez D, et al. COVID-19 and Kawasaki Disease: Novel Virus and Novel Case. Hosp Pediatr. 2020;10(6):537–40. 10.1542/hpeds.2020-0123. 10.1542/hpeds.2020-0123 32265235

[80] Vanegas Ramirez A, Efe D, Fischer M. Drug-induced vasculitis in a patient with COVID-19. J Eur Acad Dermatol Venereol. 2020;34(8):e361–2. 10.1111/jdv.16588. 10.1111/jdv.16588 PMC7267143 32378770

[81] Castelnovo L, Capelli F, Tamburello A, Faggioli PM, Mazzone A. Symmetric cutaneous vasculitis in COVID-19 pneumonia. J Eur Acad Dermatol Venereol. 2020;34(8):e362–3. 10.1111/jdv.16589. 10.1111/jdv.16589 PMC7267621 32378747

[82] Moeinzadeh F, Dezfouli M, Naimi A, Shahidi S, Moradi H (2020). Newly diagnosed glomerulonephritis during COVID-19 infection undergoing immunosuppression therapy, a case report. Iran J Kidney Dis.

[83] Galván Casas C, Català A, Carretero Hernández G, et al. Classification of the cutaneous manifestations of COVID-19: a rapid prospective nationwide consensus study in Spain with 375 cases. Br J Dermatol. 2020;183(1):71–7. 10.1111/bjd.19163. 10.1111/bjd.19163 PMC7267236 32348545

[84] de Masson A, Bouaziz JD, Sulimovic L, et al. Chilblains is a common cutaneous finding during the COVID-19 pandemic: a retrospective nationwide study from France. J Am Acad Dermatol. 2020;83(2):667–70. 10.1016/j.jaad.2020.04.161. 10.1016/j.jaad.2020.04.161 PMC7198162 32380219

[85] Kolivras A, Dehavay F, Delplace D, et al. Coronavirus (COVID-19) infection-induced chilblains: A case report with histopathologic findings. JAAD Case Rep. 2020;6(6):489–92. 10.1016/j.jdcr.2020.04.011. 10.1016/j.jdcr.2020.04.011 PMC7194989 32363225

[86] Cordoro KM, Reynolds SD, Wattier R, McCalmont TH. Clustered cases of acral perniosis: Clinical features, histopathology, and relationship to COVID-19. Pediatr Dermatol. 2020;37(3):419–23. 10.1111/pde.14227. 10.1111/pde.14227 PMC7272804 32396999

[87] Colonna C, Monzani NA, Rocchi A, Gianotti R, Boggio F, Gelmetti C. Chilblain-like lesions in children following suspected COVID-19 infection. Pediatr Dermatol. 2020;37(3):437–40. 10.1111/pde.14210. 10.1111/pde.14210 PMC7267284 32374033

[88] Bouaziz JD, Duong T, Jachiet M, et al. Vascular skin symptoms in COVID-19: a french observational study. J Eur Acad Dermatol Venereol. 2020;10.1111/jdv.16544. 10.1111/jdv.16544. 10.1111/jdv.16544 PMC7267662 32339344

[89] Andina D, Noguera-Morel L, Bascuas-Arribas M, et al. Chilblains in children in the setting of COVID-19 pandemic. Pediatr Dermatol. 2020;37(3):406–11. 10.1111/pde.14215. 10.1111/pde.14215 PMC7272985 32386460

[90] Suarez-Valle A, Fernandez-Nieto D, Diaz-Guimaraens B, Dominguez-Santas M, Carretero I, Perez-Garcia B. Acro-ischaemia in hospitalized COVID-19 patients. J Eur Acad Dermatol Venereol. 2020;10.1111/jdv.16592. 10.1111/jdv.16592. 10.1111/jdv.16592 PMC7267280 32378743

[91] Alramthan A, Aldaraji W. Two cases of COVID-19 presenting with a clinical picture resembling chilblains: first report from the Middle East. Clin Exp Dermatol. 2020;45(6):746–8. 10.1111/ced.14243. 10.1111/ced.14243 PMC7264553 32302422

[92] Zhou Y, Han T, Chen J, et al. Clinical and Autoimmune Characteristics of Severe and Critical Cases of COVID-19. Clin Transl Sci. 2020;10.1111/cts.12805. 10.1111/cts.12805. 10.1111/cts.12805 PMC7264560 32315487

[93] Zhang Y, Xiao M, Zhang S, Xia P, Cao W, Jiang W (2020). Coagulopathy and Antiphospholipid antibodies in patients with Covid-19. N Engl J Med.

[94] Galeano-Valle F, Oblitas CM, Ferreiro-Mazón MM, et al. Antiphospholipid antibodies are not elevated in patients with severe COVID-19 pneumonia and venous thromboembolism. Thromb Res. 2020;192:113–5. 10.1016/j.thromres.2020.05.017. 10.1016/j.thromres.2020.05.017 PMC7227496 32425261

[95] Harzallah I, Debliquis A, Drénou B. Lupus anticoagulant is frequent in patients with Covid-19. J Thromb Haemost. 2020;18(8):2064–5. 10.1111/jth.14867. 10.1111/jth.14867 PMC7264773 32324958

[96] Bowles L, Platton S, Yartey N, et al. Lupus Anticoagulant and Abnormal Coagulation Tests in Patients with Covid-19. N Engl J Med. 2020;383(3):288–90. 10.1056/NEJMc2013656. 10.1056/NEJMc2013656 PMC7217555 32369280

[97] Beyrouti R, Adams ME, Benjamin L, et al. Characteristics of ischaemic stroke associated with COVID-19. J Neurol Neurosurg Psychiatry. 2020;91(8):889–91. 10.1136/jnnp-2020-323586. 10.1136/jnnp-2020-323586 PMC7231545 32354768

[98] Lazarian G, Quinquenel A, Bellal M, et al. Autoimmune haemolytic anaemia associated with COVID-19 infection. Br J Haematol. 2020;190(1):29–31. 10.1111/bjh.16794. 10.1111/bjh.16794 PMC7267601 32374906

[99] Lopez C, Kim J, Pandey A, Huang T, DeLoughery TG. Simultaneous onset of COVID-19 and autoimmune haemolytic anaemia. Br J Haematol. 2020;190(1):31–2. 10.1111/bjh.16786. 10.1111/bjh.16786 PMC7267644 32369626

[100] Bomhof G, Mutsaers PGNJ, Leebeek FWG, et al. COVID-19-associated immune thrombocytopenia. Br J Haematol. 2020;190(2):e61–4. 10.1111/bjh.16850. 10.1111/bjh.16850 PMC7276755 32420612

[101] Zulfiqar AA, Lorenzo-Villalba N, Hassler P, Andrès E (2020). Immune thrombocytopenic Purpura in a patient with Covid-19. N Engl J Med.

[102] Li M, Nguyen CB, Yeung Z, Sanchez K, Rosen D, Bushan S. Evans syndrome in a patient with COVID-19. Br J Haematol. 2020;190(2):e59–61. 10.1111/bjh.16846. 10.1111/bjh.16846 PMC7276874 32420629

[103] Dogan L, Kaya D, Sarikaya T, et al. Plasmapheresis treatment in COVID-19-related autoimmune meningoencephalitis: Case series. Brain Behav Immun. 2020;87:155–8. 10.1016/j.bbi.2020.05.022. 10.1016/j.bbi.2020.05.022 PMC7204750 32389697

[104] Pilotto A, Odolini S, Masciocchi S, et al. Steroid-Responsive Encephalitis in Coronavirus Disease 2019. Ann Neurol. 2020;10.1002/ana.25783. 10.1002/ana.25783. 10.1002/ana.25783 PMC7276848 32418288

[105] Sedaghat Z, Karimi N. Guillain Barre syndrome associated with COVID-19 infection: a case report. J Clin Neurosci. 2020;76:233–5. 10.1016/j.jocn.2020.04.062. 10.1016/j.jocn.2020.04.062 PMC7158817 32312628

[106] Pease CT, Haugeberg G, Montague B, Hensor EM, Bhakta BB, Thomson W (2009). Polymyalgia rheumatica can be distinguished from late onset rheumatoid arthritis at baseline: results of a 5-yr prospective study. Rheumatology (Oxford).

[107] Chuang TY, Hunder GG, Ilstrup DM, Kurland LT (1982). Polymyalgia rheumatica: a 10-year epidemiologic and clinical study. Ann Intern Med.

[108] Dalakas MC, Hohlfeld R (2003). Polymyositis and dermatomyositis. Lancet..

[109] Tomimitsu H, Ohta A, Nagai M, Nishina M, Ishihara S, Kohsaka H (2016). Epidemiologic analysis of the clinical features of Japanese patients with polymyositis and dermatomyositis. Mod Rheumatol.

[110] Noda K, Yoshida K, Ukichi T, Furuya K, Hirai K, Kingetsu I (2017). Myalgia in patients with Dermatomyositis and Polymyositis is attributable to fasciitis rather than myositis: a retrospective study of 32 patients who underwent Histopathological examinations. J Rheumatol.

[111] Medsger TA, Rodnan GP, Moossy J, Vester JW (1968). Skeletal muscle involvement in progressive systemic sclerosis (scleroderma). Arthritis Rheum.

[112] Ranque B, Authier FJ, Le-Guern V, Pagnoux C, Berezne A, Allanore Y (2009). A descriptive and prognostic study of systemic sclerosis-associated myopathies. Ann Rheum Dis.

[113] Wallace D, Hahn B. Dubois' lupus Erythematosus. 7th ed. Philadelphia: Lippincott Williams & Wilkins; 2007.

[114] Guillevin L, Durand-Gasselin B, Cevallos R, Gayraud M, Lhote F, Callard P (1999). Microscopic polyangiitis: clinical and laboratory findings in eighty-five patients. Arthritis Rheum.

[115] Senarathna HM, Fonseka CL, Perera HAS, De Silva PUT, Weerarathna TP (2019). Severe disabling myalgia as an initial presentation of Polyarteritis Nodosa. Case Rep Rheumatol.

[116] Bahou E, Zhou L (2017). ANCA-associated vasculitis predominantly presenting with severe myalgias. Neurol Neuroimmunol Neuroinflamm.

[117] Hewlett S, Choy E, Kirwan J (2012). Furthering our understanding of fatigue in rheumatoid arthritis. J Rheumatol.

[118] Overman CL, Kool MB, Da Silva JA, Geenen R (2016). The prevalence of severe fatigue in rheumatic diseases: an international study. Clin Rheumatol.

[119] Richards HL, Herrick AL, Griffin K, Gwilliam PD, Loukes J, Fortune DG (2003). Systemic sclerosis: patients' perceptions of their condition. Arthritis Rheum.

[120] Ahn GE, Ramsey-Goldman R (2012). Fatigue in systemic lupus erythematosus. Int J Clin Rheumtol.

[121] Basu N, McClean A, Harper L, Amft EN, Dhaun N, Luqmani RA (2013). Explaining fatigue in ANCA-associated vasculitis. Rheumatology (Oxford).

[122] Grayson PC, Amudala NA, McAlear CA, Leduc RL, Shereff D, Richesson R (2013). Illness perceptions and fatigue in systemic vasculitis. Arthritis Care Res (Hoboken).

