# Supplementary material for: Rheumatic manifestations of COVID-19: a systematic review and meta-analysis
Source: BMC Rheumatol. 2020 Oct 28;4:65. doi: 10.1186/s41927-020-00165-0 (PMC7591274; doi:10.1186/s41927-020-00165-0)
Supplement: Supplementary file 1 — Additional file 1 Table S1. Quality assessment of randomized clinical trials. Table S2. Quality assessment of observational studies. Figure S1. Funnel plot. Meta-analysis of muscle pain as presenting symptom of COVID-19. Figure S2. Funnel plot. Meta-analysis of muscle pain prevalence during the course of COVID-19. Figure S3. Funnel plot. Meta-analysis of fatigue as presenting symptom of COVID-19. Figure S4. Funnel plot. Meta-analysis of fatigue prevalence during the course of COVID-19. [file 41927_2020_165_MOESM1_ESM.docx]

Table S1. Quality assessment of randomized clinical trials.

|  | Selection bias | Allocation concealment | Selective reporting | Incomplete outcome data | Blinding (participants and personnel) | Blinding (outcome assessment) | Other sources of bias |  |
| --- | --- | --- | --- | --- | --- | --- | --- | --- |
| Hu, Phytomedicine | | High | Unclear | Low | Low | Unclear | Low | Low |
| Hung, Lancet | | Low | Low | Unclear | Low | High | Unclear | Low |
| Tang, BMJ | | Low | Low | Low | Low | High | Unclear | Low |

Table S2. Quality assessment of observational studies.

|  | Representativeness of the exposed cohort | Selection of the non-exposed cohort | Ascertainment of exposure | Demonstration that outcome of interest was not present at study entry | Comparability of cohorts on the basis of the design or analysis | Assessment of outcome | Was follow-up long enough for outcomes to occur | Adequacy of follow-up of cohorts | Total quality score | Quality |
| --- | --- | --- | --- | --- | --- | --- | --- | --- | --- | --- |
| Cao J, Clin Infect Dis | X |  | X | X | X | X | X | X | 7 | Good |
| Chen J; J Infect | X |  | X | X | X | X | X | X | 7 | Good |
| Chen L; Acta Ophthalmol | X |  | X | X | X | X |  |  | 5 | Poor |
| Chen Q; Infection | X |  | X | X | X | X | X | X | 7 | Good |
| Chen R; Chest | X |  | X | X |  | X | X | X | 6 | Good |
| Chen T; Bmj | X |  | X | X | X | X | X | X | 7 | Good |
| Chen T; J Gerontol A Bio Sci Med Sci | X |  | X | X | X | X | X | X | 7 | Good |
| Chen Y; Diabetes Care | X |  | X | X |  | X | X | X | 6 | Good |
| de Masson A; J Am Acad Dermatol | X |  | X | X |  | X |  |  | 4 | Poor |
| Du R; Ann Am Thorac Soc | X |  | X | X | X | X | X | X | 7 | Good |
| Galeano-Valle F; Thromb Res | X |  | X | X |  | X |  |  | 4 | Poor |
| Galván-Casas C; Br J Dermatol | X |  | X | X |  | X | X |  | 5 | Fair |
| Garazzino S; Euro Surveill | X |  | X | X |  | X | X |  | 5 | Fair |
| Huang J; J Med Virol | X |  | X | X | X | X | X | X | 7 | Good |
| Huang R; PLoS Negl Trop Dis | X |  | X | X | X | X | X | X | 7 | Good |
| Hur K; Otolaryngol Head Neck Surg | X |  | X | X | X |  | X |  | 5 | Fair |
| Javanian M; Rom J Int Med | X |  | X | X | X | X | X |  | 6 | Good |
| Ji M; Epidemiol Infect | X |  | X | X | X | X | X | X | 7 | Good |
| Klopfenstein T; Clin Res Hepatol Gastrenterol | X |  | X | X |  |  | X |  | 4 | Poor |
| Li W; Clin Infect Dis | X |  | X | X |  |  |  | X | 4 | Poor |
| Lian J; Clin Infect Dis | X |  | X | X | X | X | X | X | 7 | Good |
| Liguori C; Brain Behav Immun | X |  | X | X | X |  | X |  | 5 | Fair |
| Liu F; J Clin Virol | X |  | X | X | X | X |  | X | 6 | Good |
| Liu Y; J Infect | X |  | X | X | X | X | X | X | 7 | Good |
| Lu Y; Pediatr Infect | X |  | X | X | X | X | X | X | 7 | Good |
| Meng Y; PLoS Pathog | X |  | X | X | X | X | X |  | 6 | Good |
| Mo P; Clin Infect Dis | X |  | X | X | X |  |  | X | 5 | Fair |
| Nowak B; Pol Arch Intern Med | X |  | X | X | X | X | X | X | 7 | Good |
| Paderno A; Int Forum Allergy Rhinol | X |  | X | X | X |  |  |  | 4 | Poor |
| Palaiodimos L; Metabolism | X |  | X | X | X | X | X | X | 7 | Good |
| Pan L; Am J Gastroenterol | X |  | X | X | X |  | X | X | 6 | Good |
| Qi L; Int J Infect Dis | X |  | X | X | X |  | X |  | 5 | Fair |
| Redd WD; Gastroenterology | X |  | X | X | X | X | X | X | 7 | Good |
| Ren D; Intensive Care Med | X |  | X | X | X | X | X | X | 7 | Good |
| Shi Q; Diabetes Care | X |  | X | X | X | X |  |  | 5 | Poor |
| Shi S; JAMA Cardiol | X |  | X | X | X | X | X | X | 7 | Good |
| Tian S; J infect | X |  | X | X | X | X | X | X | 7 | Good |
| Verdoni L; Lancet | X |  | X | X |  |  |  | X | 4 | Poor |
| Wang D; Crit Care | X |  | X | X | X |  | X | X | 6 | Good |
| Wang D; Jama | X |  | X | X | X | X | X | X | 7 | Good |
| Wang G; J Med Virol | X |  | X | X | X | X |  | X | 6 | Good |
| Wang R; Int J Infect Dis | X |  | X | X |  | X | X |  | 5 | Fair |
| Wang Z; Diabetes Res Clin Pract | X |  | X | X | X | X |  | X | 6 | Good |
| Wu C; JAMA Intern Med | X |  | X | X | X | X | X | X | 7 | Good |
| Yan Y; BMJ Open Diabetes Res Care | X |  | X | X | X | X | X |  | 6 | Good |
| Yang W; J Infect | X |  | X | X |  | X | X | X | 6 | Good |
| Yao Q; Pol Arch Intern Med | X |  | X | X | X | X | X |  | 6 | Good |
| Zhang G; J Clin Virol | X |  | X | X |  | X | X |  | 5 | Fair |
| Zhang G; J Infect Dis | X |  | X | X | X | X | X | X | 7 | Good |
| Zhang J; J Clin Virol | X |  | X | X |  | X | X |  | 5 | Fair |
| Zhang Y; Diabetes Obes Metab | X |  | X | X | X | X |  | X | 6 | Good |
| Zhang R; Eur Radiol | X |  | X | X | X |  | X | X | 6 | Good |
| Zhao W; AJR Am J Roentgenol | X |  | X | X | X |  | X |  | 5 | Fair |
| Zheng F; Eur Rev Med Pharmacol Sci | X |  | X | X | X | X | X | X | 7 | Good |
| Zheng Y; Clin Chem Lab Med | X |  | X | X |  | X | X | X | 6 | Good |
| Zhou F; Lancet | X |  | X | X | X | X | X | X | 7 | Good |
| Zhou S; Eur Radiol | X |  | X | X | X | X | X |  | 6 | Good |
| Zhou X; Clin Exp Hypertens | X |  | X | X | X |  | X | X | 6 | Good |
| Zhou Y; PloS One | X |  | X | X | X | X | X | X | 7 | Good |

Figure S1. Funnel plot. Meta-analysis of muscle pain as presenting symptom of COVID-19.

Figure S2. Funnel plot. Meta-analysis of muscle pain prevalence during the course of COVID-19.

Figure S3. Funnel plot. Meta-analysis of fatigue as presenting symptom of COVID-19.

Figure S4. Funnel plot. Meta-analysis of fatigue prevalence during the course of COVID-19.
